# Supplementary material for: Basal-to-inflammatory transition and tumor resistance via crosstalk with a pro-inflammatory stromal niche
Source: Nat Commun. 2024 Sep 17;15:8134. doi: 10.1038/s41467-024-52394-3 (PMC11408617; doi:10.1038/s41467-024-52394-3)
Supplement: Supplementary file 1 — Supplementary Information [file 41467_2024_52394_MOESM1_ESM.pdf]

a

Patient Tumors used for main figure analysis

						(Haensel et al)			(Kuonen et al)					(Yao et al)			
Patient	HuBCC1	HuBCC2	HuBCC3	HuBCC4	HuBCC5	HuBCC6	HuBCC7	HuBCC8	HuBCC9	HuBCC10	HuBCC11	HuBCC12	HuBCC13	HuBCC14	HuBCC15	HuBCC16	HuBCC17
scRNAseq	Yes	Yes	Yes	Yes	Yes	Yes	Yes	Yes	Yes	Yes	Yes	Yes	Yes	Yes	Yes	Yes	Yes
scATACseq	Yes	No	No	No	No	Yes	No	No	No	No	No	No	No	No	No	No	No
Visium	Yes	No	No	No	No	Yes	No	No	No	No	No	No	No	No	No	No	No
Tumor sample for CODEX	1	0	0	0	0	3	0	0	0	0	0	0	0	0	0	0	0

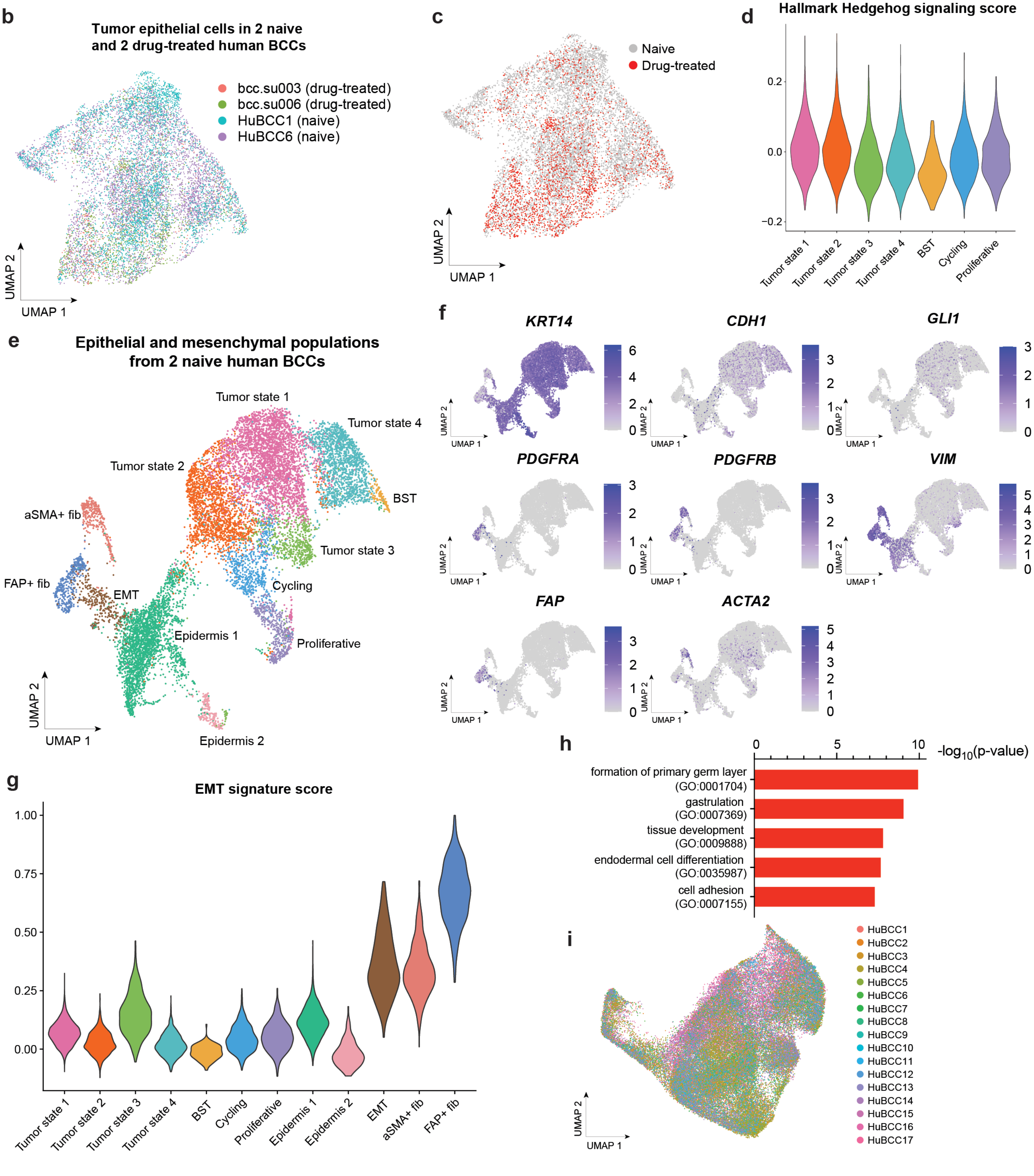

Supplementary Figure 1.

a. Summary table of patient tumor samples used for main figure analysis[1-3] and analytical methods applied to each patient tumor sample.

b. UMAP plot of scRNA-Seq data consisting of 11041 cells from four integrated human BCC tumor epithelial cells shown by the different tumor identities from Fig. 1b.

c. UMAP plot of scRNA-Seq data consisting of 11041 cells from four integrated human BCC tumor epithelial cells colored by drug treatment status from Fig. 1b.

d. Violin plot of Hallmark Hedgehog signaling signature score across tumor epithelial cells from two naïve human BCC tumors from Fig. 1e.

e. UMAP plot of scRNA-Seq data consisting of 13080 cells from epithelial and mesenchymal populations from two naive human BCC tumors, where clusters are labeled by cell types/states.

f. Feature plots of the different cell type markers for the human BCC tumors in Supplementary Figure 1e.

g. Violin plot of EMT signature score across epithelial and mesenchymal populations from two naive human BCC tumors from Supplementary Figure 1e.

h. Gene Ontology (GO) Term analysis of TS3 cluster marker genes shown in Fig. 1f. One-sided Fisher's exact test was used to compute p-value statistics for each GO Term. P-values were corrected for multiple testing using Benjamini and Hochberg method.

i. UMAP plot of scRNA-Seq data consisting of 66429 cells from 17 integrated human BCC tumor epithelium shown by the different tumor identities from Fig. 1g.

Source data are provided as a Source Data file.

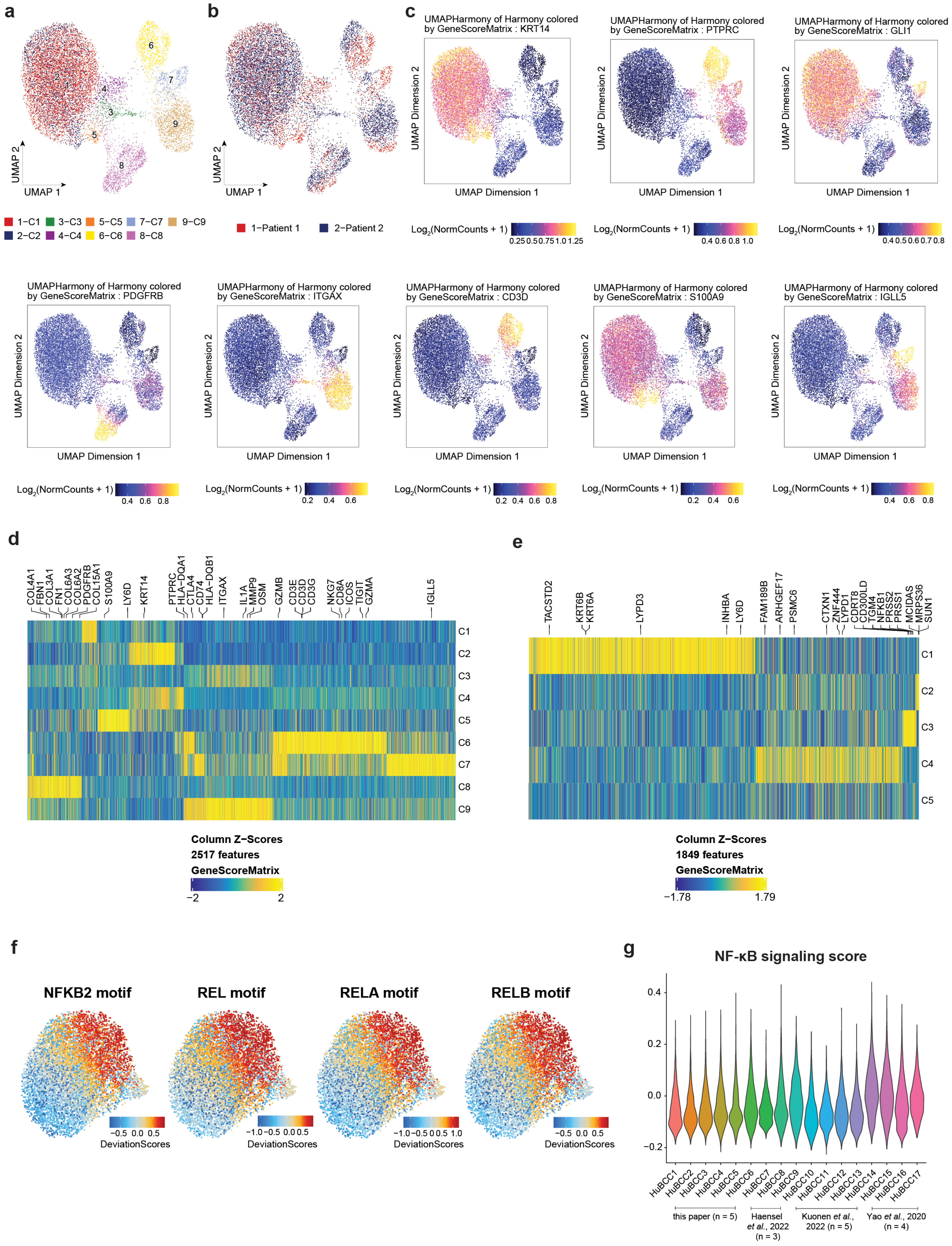

a
b
c
UMAPHarmony of Harmony colored by GeneScoreMatrix : KRT14
UMAPHarmony of Harmony colored by GeneScoreMatrix : PTPRC
UMAPHarmony of Harmony colored by GeneScoreMatrix : GLI1
UMAPHarmony of Harmony colored by GeneScoreMatrix : PDGFRB
UMAPHarmony of Harmony colored by GeneScoreMatrix : ITGAX
UMAPHarmony of Harmony colored by GeneScoreMatrix : CD3D
UMAPHarmony of Harmony colored by GeneScoreMatrix : S100A9
UMAPHarmony of Harmony colored by GeneScoreMatrix : IGLL5
UMAP 1
UMAP 2
UMAP Dimension 1
UMAP Dimension 2
Log2(NormCounts + 1)
1−C1 3−C3 5−C5 7−C7 9−C9
2−C2 4−C4 6−C6 8−C8
1−Patient 1 2−Patient 2
d
e
Column Z−Scores
2517 features
GeneScoreMatrix
−2 2
Column Z−Scores
1849 features
GeneScoreMatrix
−1.78 1.79
f
NFKB2 motif
REL motif
RELA motif
RELB motif
DeviationScores
g
NF-κB signaling score
HuBCC1 HuBCC2 HuBCC3 HuBCC4 HuBCC5 HuBCC6 HuBCC7 HuBCC8 HuBCC9 HuBCC10 HuBCC11 HuBCC12 HuBCC13 HuBCC14 HuBCC15 HuBCC16 HuBCC17
this paper (n = 5)
Haensel et al., 2022 (n = 3)
Kuonen et al., 2022 (n = 5)
Yao et al., 2020 (n = 4)

Supplementary Figure 2.

a. UMAP plot of Harmony corrected scATAC-Seq data consisting of 13514 cells from two merged human BCC tumor samples displaying all clusters.

b. UMAP plot of Harmony corrected scATAC-Seq data consisting of 13514 cells from two merged human BCC tumor samples colored by tumor identities.

c. Feature plots of the GeneScoreMatrix for different cell type markers for the merged scATAC-Seq data shown in Supplementary Figure 2a.

d. Heatmap of all the major cell type features associated with each cluster of the merged scATAC-Seq data shown in Supplementary Figure 2a.

e. Heatmap of marker genes associated with each of the 5 tumor epithelial clusters of the merged scATAC-Seq data shown in Fig. 2c.

f. Feature plots of the motif DeviationMatrix for NFKB family motifs in scATAC-Seq data with the same UMAP projection from Fig. 2c. The higher the deviation score indicates greater enrichment of the TF motifs.

g. Violin plot of NFkB signaling score across tumor epithelial cells from 17 human BCC patients[1-3] from Supplementary Figure 1i.

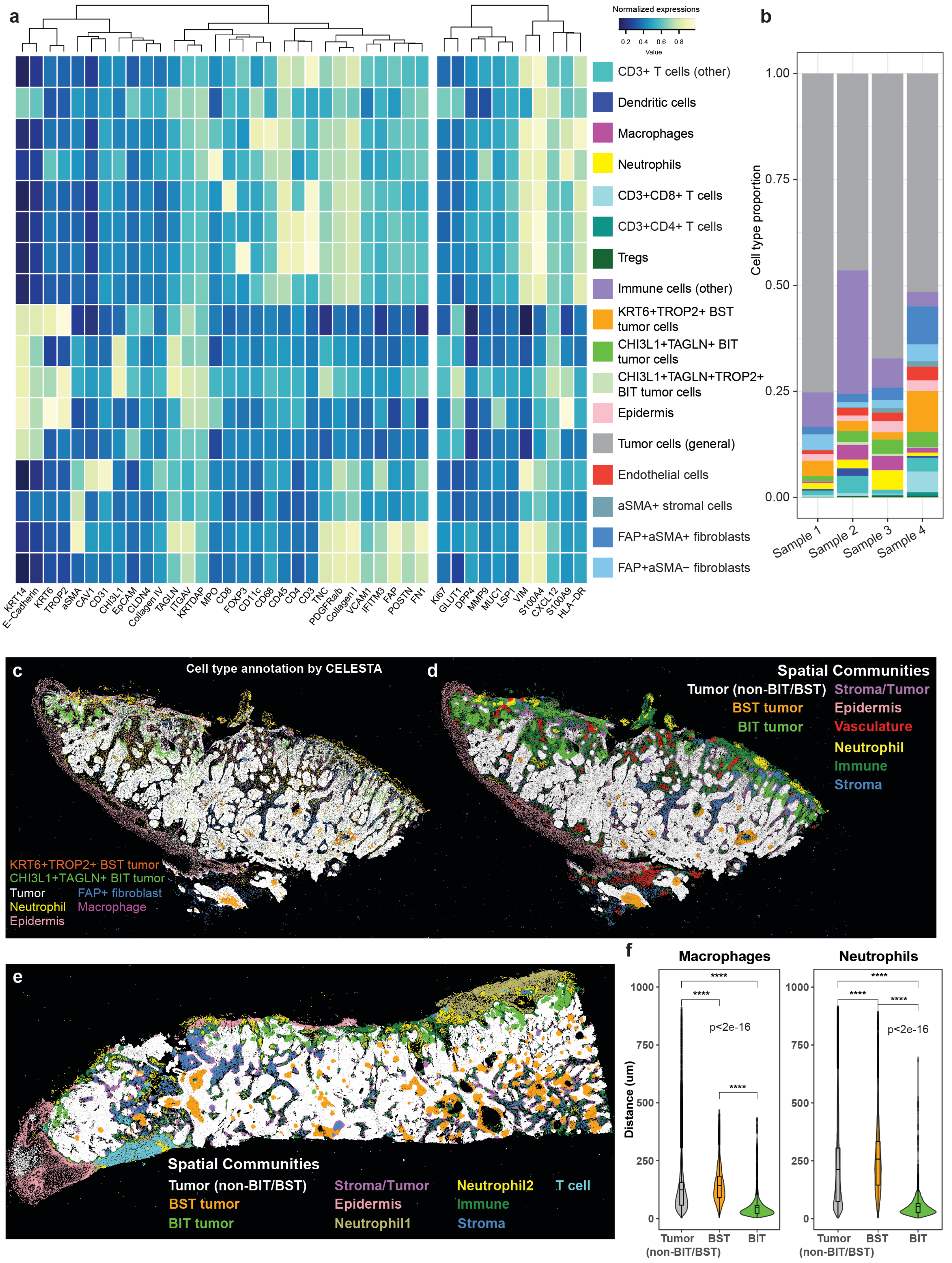
a
Normalized expressions
0.2 0.4 0.6 0.8
Value
CD3+ T cells (other)
Dendritic cells
Macrophages
Neutrophils
CD3+CD8+ T cells
CD3+CD4+ T cells
Tregs
Immune cells (other)
KRT6+TROP2+ BST tumor cells
CHI3L1+TAGLN+ BIT tumor cells
CHI3L1+TAGLN+TROP2+ BIT tumor cells
Epidermis
Tumor cells (general)
Endothelial cells
aSMA+ stromal cells
FAP+aSMA+ fibroblasts
FAP+aSMA− fibroblasts
KRT14 E−Cadherin KRT6 TROP2 aSMA CAV1 CD31 CHI3L1 EpCAM CLDN4 Collagen IV TAGLN ITGAV KRTDAP MPO CD8 FOXP3 CD11c CD68 CD45 CD4 CD3 TNC PDGFRa/b Collagen I VCAM1 IFITM3 FAP POSTN FN1
Ki67 GLUT1 DPP4 MMP9 MUC1 LSP1 VIM S100A4 CXCL12 S100A9 HLA−DR
b
Cell type proportion
1.00
0.75
0.50
0.25
0.00
Sample 1 Sample 2 Sample 3 Sample 4
c
Cell type annotation by CELESTA
KRT6+TROP2+ BST tumor
CHI3L1+TAGLN+ BIT tumor
Tumor
FAP+ fibroblast
Neutrophil
Macrophage
Epidermis
d
Spatial Communities
Tumor (non-BIT/BST)
Stroma/Tumor
BST tumor
Epidermis
BIT tumor
Vasculature
Neutrophil
Immune
Stroma
e
Spatial Communities
Tumor (non-BIT/BST)
Stroma/Tumor
Neutrophil2
T cell
BST tumor
Epidermis
Immune
BIT tumor
Neutrophil1
Stroma
f
Macrophages
Neutrophils
Distance (um)
1000
750
500
250
0
****
p<2e-16
Tumor (non-BIT/BST)
BST
BIT

Supplementary Figure 3.

a. Heatmap showing expression levels of all 41 protein markers in the CODEX panel in each of the 17 CELESTA-assigned cell types.

b. Cell type composition plot displaying proportions of 17 CELESTA-assigned cell types for each sample.

c. Representative whole slide image of another human BCC tumor depicting cell types annotated by CELESTA.

d. Identification of nine distinct spatial communities in another human BCC tumor based on CELESTA-assigned cell types and their respective frequencies within each spatial community.

e. Identification of ten distinct spatial communities in another human BCC tumor based on CELESTA-assigned cell types and their respective frequencies within each spatial community.

f. Violin plots depicting distributions of the distances from the three tumor types: tumor (non-BIT/BST), BST, and BIT, to nearest macrophages and neutrophils across all four CODEX samples. The center corresponds to the mean value. The lower and upper hinges correspond to the 25th and 75th percentiles. The upper whisker extends from hinge to the largest value no further than 1.5*IQR (Inter-Quartile Range). The lower whisker extends from lower hinge to the smallest value at most 1.5*IQR. P-values were calculated using two-sided independent sample T test (****p<2e-16).

Source data are provided as a Source Data file.

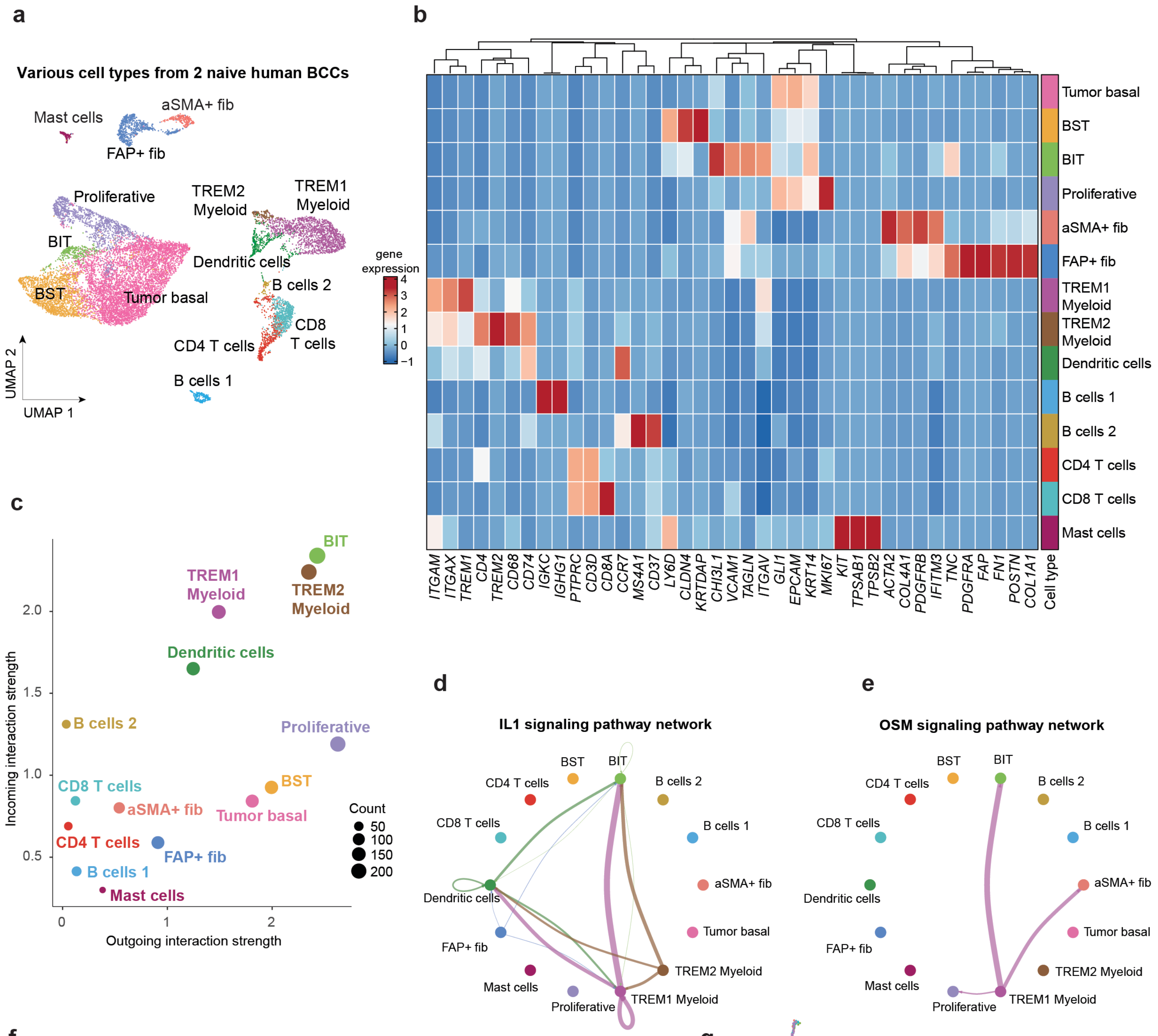

f

Patient Tumor	HuBCC1	HuBCC6	HuBCC2	HuBCC3
% of BIT Cells	5.8	4.6	1.8	1.6
BIT Status	High	High	Low	Low

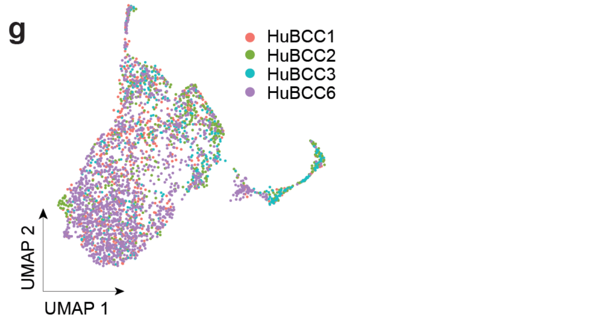

Supplementary Figure 4.

a. UMAP plot of 14047 cells from two naive human BCC tumors for CellChat analysis.

b. Heatmap of all the major cell type cluster markers from human BCC tumors in Supplementary Figure 4a.

c. Scatter plot depicting the dominant senders and receivers of outgoing and incoming interactions respectively in a 2D space based on signaling role analysis on the aggregated cell-cell communication network from all signaling pathways from data in Supplementary Figure 4a.

d. IL1 signaling pathway network across various cell types in Supplementary Figure 4a.

e. OSM signaling pathway network across various cell types in Supplementary Figure 4a.

f. Summary table on proportions of BIT cells in tumor epithelial populations across 4 naive human BCC tumors. Tumors with greater than 3% BIT cells among tumor epithelial populations are labelled BIT high, while tumors with fewer than 3% BIT cells among tumor epithelial populations are labelled BIT low.

g. UMAP plot of scRNA-Seq data of myeloid cell populations consisting of 3144 cells from four naïve human BCC tumors in Fig. 4g colored by patient.

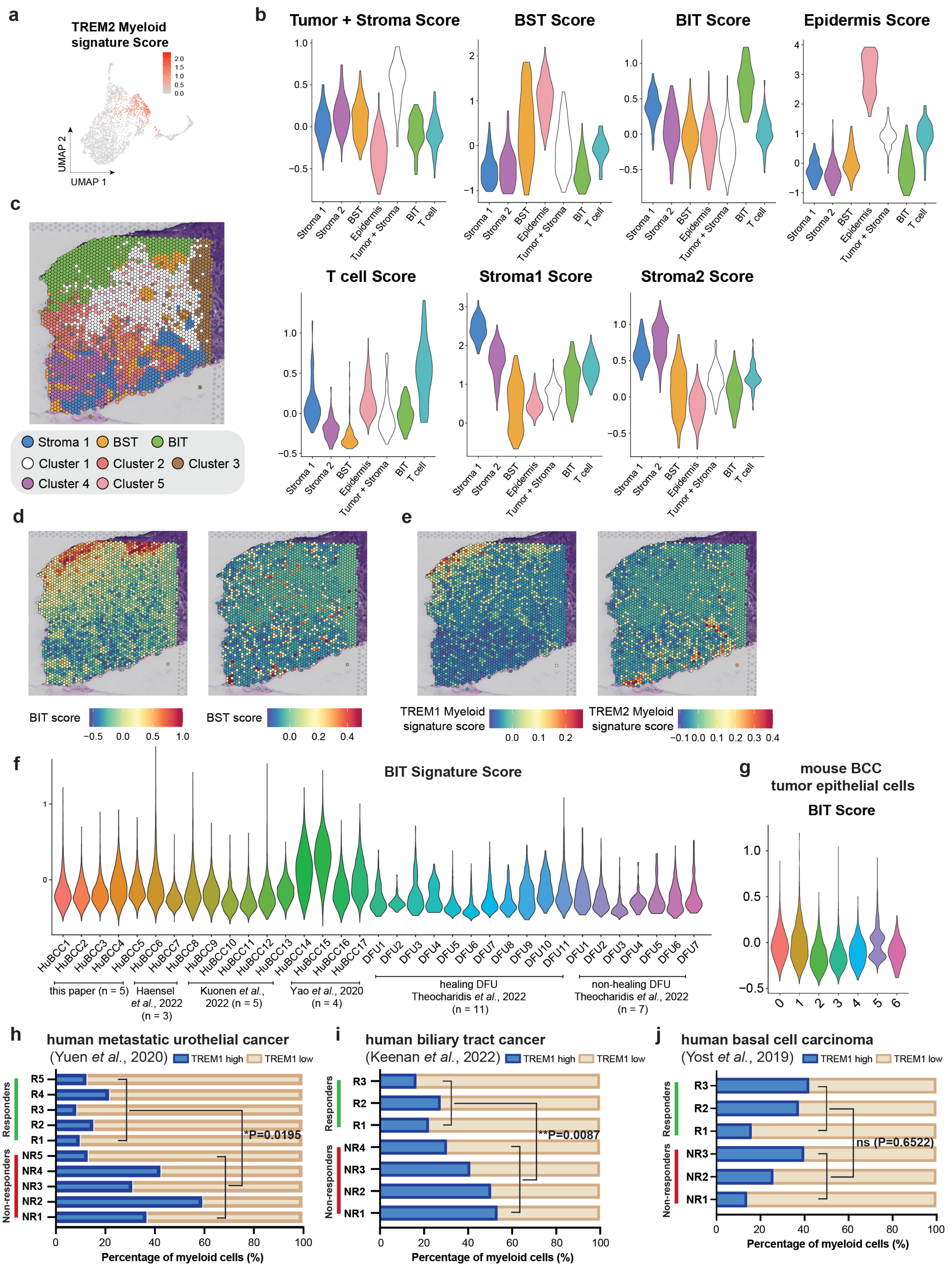
a
TREM2 Myeloid signature Score
UMAP 1
UMAP 2
b
Tumor + Stroma Score
BST Score
BIT Score
Epidermis Score
T cell Score
Stroma1 Score
Stroma2 Score
Stroma 1
Stroma 2
BST
Epidermis
Tumor + Stroma
BIT
T cell
c
Stroma 1
BST
BIT
Cluster 1
Cluster 2
Cluster 3
Cluster 4
Cluster 5
d
BIT score
BST score
e
TREM1 Myeloid signature score
TREM2 Myeloid signature score
f
BIT Signature Score
this paper (n = 5)
Haensel et al., 2022 (n = 3)
Kuonen et al., 2022 (n = 5)
Yao et al., 2020 (n = 4)
healing DFU Theocharidis et al., 2022 (n = 11)
non-healing DFU Theocharidis et al., 2022 (n = 7)
g
mouse BCC tumor epithelial cells
BIT Score
h
human metastatic urothelial cancer (Yuen et al., 2020)
TREM1 high
TREM1 low
Responders
Non-responders
*P=0.0195
Percentage of myeloid cells (%)
i
human biliary tract cancer (Keenan et al., 2022)
**P=0.0087
j
human basal cell carcinoma (Yost et al., 2019)
ns (P=0.6522)

Supplementary Figure 5.

a. Feature plot of TREM2 myeloid signature gene scoring across myeloid cell populations from four naïve human BCC tumors in Fig. 4g.

b. Scoring of the different Visium spatial Seurat clusters with cluster marker gene signatures to identify spatial Seurat populations in Fig. 5b.

c. Spatial DimPlot of Visium spatial Seurat clusters from another human BCC tumor.

d. Spatial Seurat gene signature scoring for BIT signature and BST signature on human BCC tumor in Supplementary Figure 5c.

e. Spatial Seurat gene signature scoring for TREM1 myeloid signature and TREM2 myeloid signature on human BCC tumor in Supplementary Figure 5c.

f. Violin plot of BIT signature gene scoring across tumor epithelial cells from 17 BCC patients[1-3] and epithelial cells from 18 diabetic foot ulcer patients[4].

g. Violin plot of BIT signature gene scoring across tumor epithelial cells from four mouse BCC tumors.

h. Percentages of myeloid with high and low TREM1 myeloid signature scores in the pretreatment peripheral blood of metastatic urothelial carcinoma patients resistant (n = 5 patients) or sensitive (n = 5 patients) to anti-PD-L1 immune checkpoint blockade therapy[5]. P-value calculated using two-sided independent sample T test (*P<0.05).

i. Percentages of myeloid with high and low TREM1 myeloid signature scores in the pre- and post-treatment peripheral blood of biliary tract cancer patients resistant (n = 4 patients) or sensitive (n = 3 patients) to anti-PD-1 immune checkpoint blockade therapy[6]. P-value calculated using two-sided independent sample T test (**P<0.01).

j. Percentages of tumor-associated myeloid cells with high and low TREM1 myeloid signature scores in tumors of basal cell carcinoma patients resistant (n = 3 patients) or sensitive (n = 3 patients) to anti-PD-1 immune checkpoint blockade therapy[7]. P-value calculated using two-sided independent sample T test (ns, P=0.6522).

Source data are provided as a Source Data file.

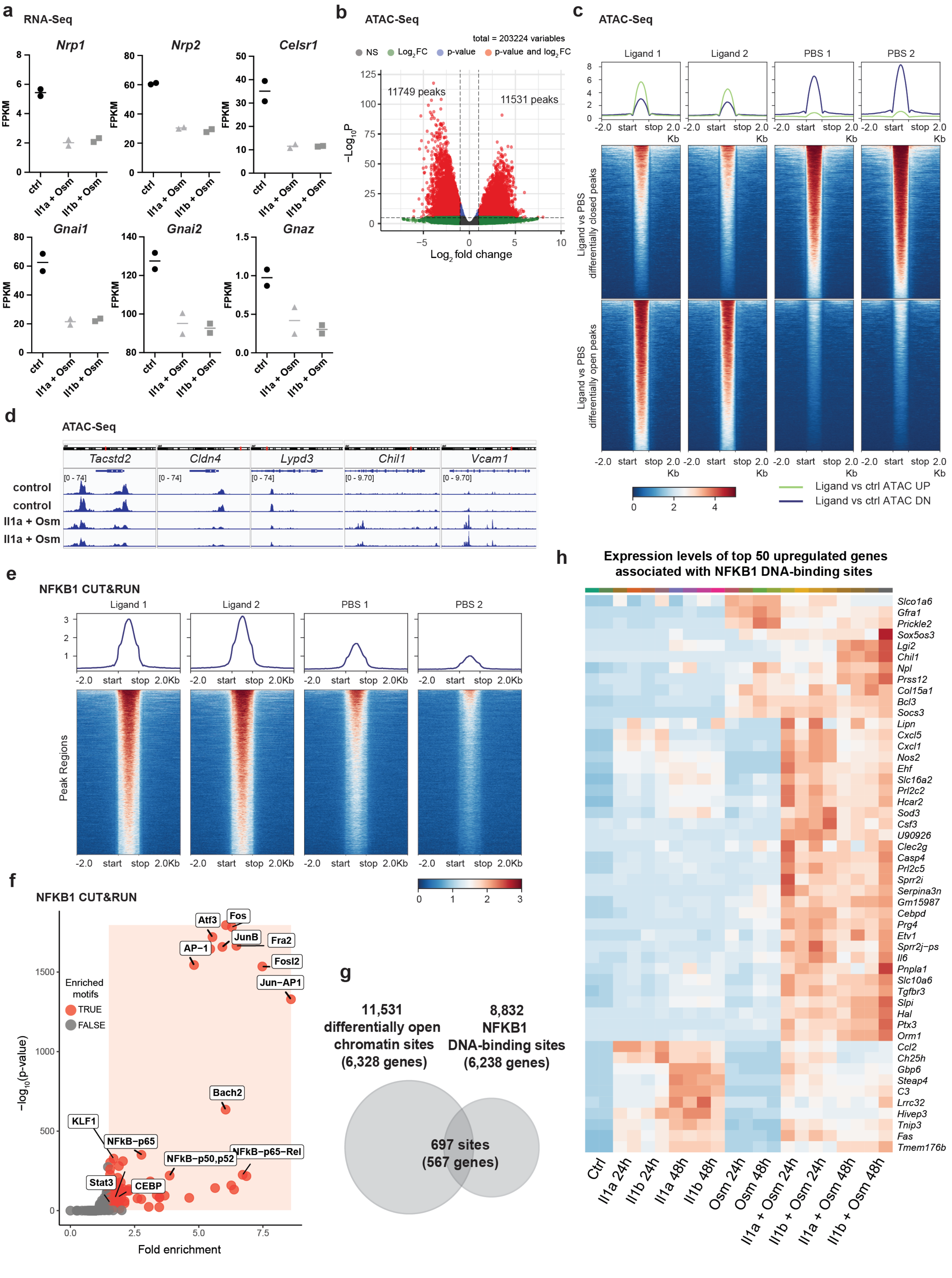
a
RNA-Seq
Nrp1
Nrp2
Celsr1
Gnai1
Gnai2
Gnaz
FPKM
ctrl
Il1a + Osm
Il1b + Osm
b
ATAC-Seq
total = 203224 variables
NS
Log2 FC
p-value
p-value and log2 FC
11749 peaks
11531 peaks
−Log10 P
Log2 fold change
c
ATAC-Seq
Ligand 1
Ligand 2
PBS 1
PBS 2
Ligand vs PBS differentially closed peaks
Ligand vs PBS differentially open peaks
-2.0 start stop 2.0 Kb
Ligand vs ctrl ATAC UP
Ligand vs ctrl ATAC DN
d
ATAC-Seq
Tacstd2
Cldn4
Lypd3
Chil1
Vcam1
[0 - 74]
[0 - 9.70]
control
control
Il1a + Osm
Il1a + Osm
e
NFKB1 CUT&RUN
Ligand 1
Ligand 2
PBS 1
PBS 2
Peak Regions
-2.0 start stop 2.0Kb
f
NFKB1 CUT&RUN
Enriched motifs
TRUE
FALSE
Atf3
Fos
JunB
Fra2
AP−1
Fosl2
Jun−AP1
Bach2
KLF1
NFkB−p65
NFkB−p50,p52
NFkB−p65−Rel
Stat3
CEBP
−log10(p-value)
Fold enrichment
g
11,531 differentially open chromatin sites (6,328 genes)
8,832 NFKB1 DNA-binding sites (6,238 genes)
697 sites (567 genes)
h
Expression levels of top 50 upregulated genes associated with NFKB1 DNA-binding sites
Slco1a6
Gfra1
Prickle2
Sox5os3
Lgi2
Chil1
Npl
Prss12
Col15a1
Bcl3
Socs3
Lipn
Cxcl5
Cxcl1
Nos2
Ehf
Slc16a2
Prl2c2
Hcar2
Sod3
Csf3
U90926
Clec2g
Casp4
Prl2c5
Sprr2i
Serpina3n
Gm15987
Cebpd
Prg4
Etv1
Sprr2j−ps
Il6
Pnpla1
Slc10a6
Tgfbr3
Slpi
Hal
Ptx3
Orm1
Ccl2
Ch25h
Gbp6
Steap4
C3
Lrrc32
Hivep3
Tnip3
Fas
Tmem176b
Ctrl
Il1a 24h
Il1b 24h
Il1a 48h
Il1b 48h
Osm 24h
Osm 48h
Il1a + Osm 24h
Il1b + Osm 24h
Il1a + Osm 48h
Il1b + Osm 48h

Supplementary Figure 6.

a. FPKM levels of hedgehog component genes upon 24-hour Il1a + Osm or Il1b + Osm treatment against control (n = 2 biological replicates).

b. Volcano plot of differentially open and closed peaks upon 48-hour Il1a + Osm treatment against control from ATAC-Seq data (n = 2 biological replicates). A total of 203224 peaks were included in the differential analysis. Peaks with Log_2fold change > 1 or < -1 and p-value < 0.00001 are marked in red.

c. Heatmap showing genome wide changes in chromatin accessibility upon Il1a + Osm treatment versus PBS control (n = 2 biological replicates).

d. Example tracks of chromatin accessibility at BST and BIT tumor marker gene loci upon 48-hour Il1a + Osm treatment against control (n = 2 biological replicates).

e. Heatmap showing genome wide changes in NFKB1 DNA binding upon Il1a + Osm treatment versus PBS control (n = 2 biological replicates).

f. Motif enrichment analysis on NFKB1 DNA-binding sites upon 48-hour Il1a + Osm treatment (n = 2 biological replicates). Significantly enriched motifs (fold enrichment ≥ 1.5 and $-\log_{10}$(p-value) ≥ 3) are highlighted in red. A total of 436 known motifs from HOMER Motif Database were included in the motif analysis. P-values were calculated using HOMER analysis pipeline. P-values were corrected for multiple testing using Benjamini and Hochberg method.

g. Venn diagram showing the number of peaks (and associated genes) that overlap between differentially open chromatin sites from ATAC-Seq data and NFKB1 DNA-binding sites from CUT&RUN sequencing data.

h. Heatmap showing RNA expression levels of top 50 upregulated genes associated with NFKB1 DNA-binding sites under various ligand treatment conditions (n = 2 biological replicates).

Source data are provided as a Source Data file.

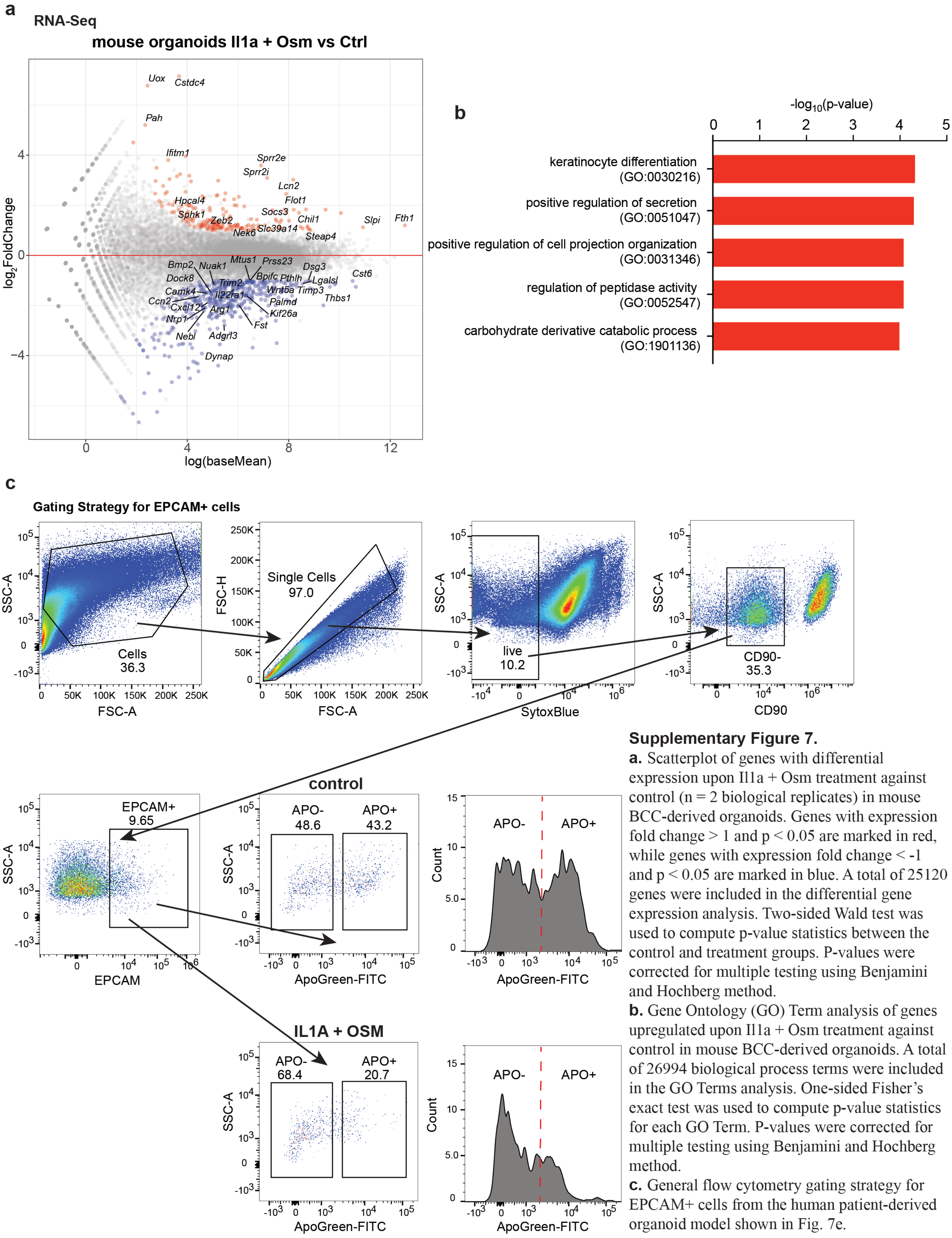

Supplementary Figure 7.
a. Scatterplot of genes with differential expression upon Il1a + Osm treatment against control (n = 2 biological replicates) in mouse BCC-derived organoids. Genes with expression fold change > 1 and p < 0.05 are marked in red, while genes with expression fold change < -1 and p < 0.05 are marked in blue. A total of 25120 genes were included in the differential gene expression analysis. Two-sided Wald test was used to compute p-value statistics between the control and treatment groups. P-values were corrected for multiple testing using Benjamini and Hochberg method.
b. Gene Ontology (GO) Term analysis of genes upregulated upon Il1a + Osm treatment against control in mouse BCC-derived organoids. A total of 26994 biological process terms were included in the GO Terms analysis. One-sided Fisher's exact test was used to compute p-value statistics for each GO Term. P-values were corrected for multiple testing using Benjamini and Hochberg method.
c. General flow cytometry gating strategy for EPCAM+ cells from the human patient-derived organoid model shown in Fig. 7e.

Source data are provided as a Source Data file.

Supplementary References

1. Haensel, D., Gaddam, S., Li, N.Y., Gonzalez, F., Patel, T., Cloutier, J.M., Sarin, K.Y., Tang, J.Y., Rieger, K.E., Aasi, S.Z., *et al.* (2022). LY6D marks pre-existing resistant basosquamous tumor subpopulations. Nat Commun *13*. 10.1038/s41467-022-35020-y.

2. Yerly, L., Pich-Bavastro, C., Di Domizio, J., Wyss, T., Tissot-Renaud, S., Cangkrama, M., Gilliet, M., Werner, S., and Kuonen, F. (2022). Integrated multi-omics reveals cellular and molecular interactions governing the invasive niche of basal cell carcinoma. Nat Commun *13*. 10.1038/s41467-022-32670-w.

3. Yao, C.D., Haensel, D., Gaddam, S., Patel, T., Atwood, S.X., Sarin, K.Y., Whitson, R.J., McKellar, S., Shankar, G., Aasi, S., *et al.* (2020). AP-1 and TGFß cooperativity drives non-canonical Hedgehog signaling in resistant basal cell carcinoma. Nat Commun *11*. 10.1038/s41467-020-18762-5.

4. Theocharidis, G., Thomas, B.E., Sarkar, D., Mumme, H.L., Pilcher, W.J.R., Dwivedi, B., Sandoval-Schaefer, T., Sîrbulescu, R.F., Kafanas, A., Mezghani, I., *et al.* (2022). Single cell transcriptomic landscape of diabetic foot ulcers. Nat Commun *13*. 10.1038/s41467-021-27801-8.

5. Yuen, K.C., Liu, L.F., Gupta, V., Madireddi, S., Keerthivasan, S., Li, C., Rishipathak, D., Williams, P., Kadel, E.E., Koeppen, H., *et al.* (2020). High systemic and tumor-associated IL-8 correlates with reduced clinical benefit of PD-L1 blockade. Nat Med *26*, 693–698. 10.1038/s41591-020-0860-1.

6. Keenan, B.P., McCarthy, E.E., Ilano, A., Yang, H., Zhang, L., Allaire, K., Fan, Z., Li, T., Lee, D.S., Sun, Y., *et al.* (2022). Circulating monocytes associated with anti-PD-1 resistance in human biliary cancer induce T cell paralysis. Cell Rep *40*. 10.1016/j.celrep.2022.111384.

7. Yost, K.E., Satpathy, A.T., Wells, D.K., Qi, Y., Wang, C., Kageyama, R., McNamara, K.L., Granja, J.M., Sarin, K.Y., Brown, R.A., *et al.* (2019). Clonal replacement of tumor-specific T cells following PD-1 blockade. Nat Med *25*, 1251–1259. 10.1038/s41591-019-0522-3.
