# Supplementary material for: Basal-to-inflammatory transition and tumor resistance via crosstalk with a pro-inflammatory stromal niche
Source: Nat Commun. 2024 Sep 17;15:8134. doi: 10.1038/s41467-024-52394-3 (PMC11408617; doi:10.1038/s41467-024-52394-3)
Supplement: Supplementary file 3 — Description of Additional Supplementary Files [file 41467_2024_52394_MOESM3_ESM.docx]

**Description of Additional Supplementary Files**

File Name: Supplementary Data 1

Description: CODEX antibody clones and barcode information. Related to Figure 3a.

File Name: Supplementary Data 2

Description: CODEX antibody titration and multiplexed imaging cycle information. Related to Figure 3a.

File Name: Supplementary Data 3

Description: Cell type signature matrix used for cell type annotation by CELESTA. Related to Figure 3d and Supplementary figures 3a-c.

File Name: Supplementary Data 4

Description: Top 100 cluster marker genes for myeloid populations from 4 naïve human BCC tumors. Related to Figure 5a.

File Name: Supplementary Data 5

Description: Top 100 upregulated genes in mouse BCC tumor cell line ASZ-001 upon 48-hour Il1a + Osm treatment against control. Related to Figure 6d.

File Name: Supplementary Data 6

Description: List of overlapped peaks (and associated genes) between ATAC-Seq data and CUT&RUN sequencing data upon 48- hour Il1a + Osm treatment against control. Related to Supplementary Figure 6g.

File Name: Supplementary Data 7

Description: List of upregulated genes associated with NFKB1 DNA-binding sites in mouse BCC tumor cell line ASZ-001 upon 48-hour Il1a + Osm treatment against control. Related to Supplementary figure 6h.
