# Supplementary material for: Basal-to-inflammatory transition and tumor resistance via crosstalk with a pro-inflammatory stromal niche
Source: Nat Commun. 2024 Sep 17;15:8134. doi: 10.1038/s41467-024-52394-3 (PMC11408617; doi:10.1038/s41467-024-52394-3)
Supplement: Supplementary file 11 — Reporting Summary [file 41467_2024_52394_MOESM11_ESM.pdf]

nature portfolio

Corresponding author(s): Anthony Oro
Last updated by author(s): Aug 21, 2024

Reporting Summary

Nature Portfolio wishes to improve the reproducibility of the work that we publish. This form provides structure for consistency and transparency in reporting. For further information on Nature Portfolio policies, see our Editorial Policies and the Editorial Policy Checklist.

Statistics

For all statistical analyses, confirm that the following items are present in the figure legend, table legend, main text, or Methods section.

n/a	Confirmed	
☐	☒	The exact sample size (*n*) for each experimental group/condition, given as a discrete number and unit of measurement
☐	☒	A statement on whether measurements were taken from distinct samples or whether the same sample was measured repeatedly
☐	☒	The statistical test(s) used AND whether they are one- or two-sided<br>*Only common tests should be described solely by name; describe more complex techniques in the Methods section.*
☒	☐	A description of all covariates tested
☐	☒	A description of any assumptions or corrections, such as tests of normality and adjustment for multiple comparisons
☐	☒	A full description of the statistical parameters including central tendency (e.g. means) or other basic estimates (e.g. regression coefficient) AND variation (e.g. standard deviation) or associated estimates of uncertainty (e.g. confidence intervals)
☐	☒	For null hypothesis testing, the test statistic (e.g. *F*, *t*, *r*) with confidence intervals, effect sizes, degrees of freedom and *P* value noted<br>*Give P values as exact values whenever suitable.*
☒	☐	For Bayesian analysis, information on the choice of priors and Markov chain Monte Carlo settings
☒	☐	For hierarchical and complex designs, identification of the appropriate level for tests and full reporting of outcomes
☒	☐	Estimates of effect sizes (e.g. Cohen's *d*, Pearson's *r*), indicating how they were calculated

Our web collection on statistics for biologists contains articles on many of the points above.

Software and code

Policy information about availability of computer code

Data collection	The various commercial/open source software was used for data collection: BD FACSDiva Version 8.0.1, Leica LAS X Version 3.7.6, Illumina NextSeq and NovaSeq System Suite, CODEX Instrument Manager (CIM, Version 1.30) and the Keyence BZ-X810 software.
Data analysis	The various commercial/open source software was used for data analysis: Seurat Version 4.2.1.9001, FIJI Version 2.1.0, 10x Genomics CellRanger Version 3.1.0, 10x Genomics SpaceRanger Version 1.3.1, R Version 4.1.2, CODEX Multiplex Analysis Viewer, Bowtie2 Version 2.3.4.1, Samtools Version 0.1.18, DESeq2 Version 1.34.0, edgeR Version 3.36.0, limma Version 3.50.0, FlowJo Version 10.6, CellChat Version 1.1.3, HOMER Version 4.11, Graphpad Prism Version 10.1.0, Monocle 3 Version 0.2.3.0, ArchR Version 1.0.1, Mesmer, CELESTA Version 0.0.0.9000, GEPIA, MACS2 Version 2.27.1, and Integrative Genomics Viewer Version 2.14.0.

For manuscripts utilizing custom algorithms or software that are central to the research but not yet described in published literature, software must be made available to editors and reviewers. We strongly encourage code deposition in a community repository (e.g. GitHub). See the Nature Portfolio guidelines for submitting code & software for further information.

Data

Policy information about availability of data

All manuscripts must include a data availability statement. This statement should provide the following information, where applicable:

- Accession codes, unique identifiers, or web links for publicly available datasets
- A description of any restrictions on data availability
- For clinical datasets or third party data, please ensure that the statement adheres to our policy

The human-specific sequencing data generated from this study (scRNA-Seq, scATAC-Seq and Visium spatial transcriptomics data) have been deposited in the dbGaP database under the accession code phs003437.v1.p1 [http://www.ncbi.nlm.nih.gov/projects/gap/cgi-bin/study.cgi?study_id=phs003437.v1.p1]. As these are raw sequencing datasets for human patients, dbGaP ensures that only authorized researchers such as tenure-track professors or senior scientists, working on similar studies with appropriate approvals can access and analyze it. Principal investigators interested in the raw datasets should submit a request for access to dbGAP study. Investigator's institutional Signing Official and NIH Data Access Committee need to approve the request to obtain access, and the process might take 2-4 weeks. Once the request is approved, the investigator will be allowed to access the dataset for one year, with the option to renew. The mouse-specific sequencing data generated from this study have been deposited in NCBI Gene Expression Omnibus database under the accession code GSE248314 [https://www.ncbi.nlm.nih.gov/geo/query/acc.cgi?acc=GSE248314]. Previous human BCC scRNA-Seq data and scATAC-Seq data used in this study are available in dbGaP database under the accession code phs003103.v1.p1 [https://www.ncbi.nlm.nih.gov/projects/gap/cgi-bin/study.cgi?study_id=phs003103.v1.p1]. The resistant human BCC scRNA-Seq publicly available data used in this study are available in the NCBI Gene Expression Omnibus under the accession code GSE123814 [https://www.ncbi.nlm.nih.gov/geo/query/acc.cgi?acc=GSE123814]. Additional human BCC scRNA-Seq datasets used in this study are available in Gene Expression Omnibus database under accession codes GSE181907 [https://www.ncbi.nlm.nih.gov/geo/query/acc.cgi?acc=GSE181907] and GSE141526 [https://www.ncbi.nlm.nih.gov/geo/query/acc.cgi?acc=GSE141526]. The inflammatory skin diseases scRNA-Seq publicly available data used in this study are available in ArrayExpress under the accession code E-MTAB-8142 [www.ebi.ac.uk/arrayexpress/experiments/E-MTAB-8142]. The alopecia areata scRNA-Seq publicly available data used in this study are available in the NCBI Gene Expression Omnibus under the accession code GSE212450 [https://www.ncbi.nlm.nih.gov/geo/query/acc.cgi?acc=GSE212450]. The colorectal cancer scRNA-Seq publicly available data used in this study are available in the NCBI Gene Expression Omnibus under the accession code GSE132465 [https://www.ncbi.nlm.nih.gov/geo/query/acc.cgi?acc=GSE132465]. The pan-cancer scRNA-Seq publicly available data used in this study are available in the NCBI Gene Expression Omnibus under the accession code GSE154763 [https://www.ncbi.nlm.nih.gov/geo/query/acc.cgi?acc=GSE154763]. The diabetic foot ulcer scRNA-Seq publicly available data used in this study are available in the NCBI Gene Expression Omnibus under the accession code GSE165816 [https://www.ncbi.nlm.nih.gov/geo/query/acc.cgi?acc=GSE165816]. The human metastatic urothelial cancer scRNA-Seq publicly available data used in this study are available in the NCBI Gene Expression Omnibus under the accession code GSE145281 [https://www.ncbi.nlm.nih.gov/geo/query/acc.cgi?acc=GSE145281]. The human biliary tract cancer CITE-Seq publicly available data used in this study are available in the NCBI Gene Expression Omnibus under the accession code GSE210067 [https://www.ncbi.nlm.nih.gov/geo/query/acc.cgi?acc=GSE210067]. Raw CODEX images are available upon request to corresponding authors due to the huge file sizes of raw data files. The remaining data are available within the Article, Supplementary Information and Source Data file. Source Data are provided with this paper.

Research involving human participants, their data, or biological material

Policy information about studies with human participants or human data. See also policy information about sex, gender (identity/presentation), and sexual orientation and race, ethnicity and racism.

Reporting on sex and gender	All patient samples have been completely de-identified as per IRB protocols and we do not have this information.
Reporting on race, ethnicity, or other socially relevant groupings	All patient samples have been completely de-identified as per IRB protocols and we do not have this information.
Population characteristics	All patient samples have been completely de-identified as per IRB protocols and we do not have this information.
Recruitment	Patients are asked by physician if they would like to participate at time. There is no obvious self-selection bias or other biases that are present.
Ethics oversight	All patient samples were obtained through written informed consent and subsequently de-identified. All protocols for sample acquisition and usage are in accordance with the reviewed protocol by the Stanford University Institutional Review Board, protocol #18325 (Stanford, CA).

Note that full information on the approval of the study protocol must also be provided in the manuscript.

Field-specific reporting

Please select the one below that is the best fit for your research. If you are not sure, read the appropriate sections before making your selection.

[x] Life sciences [ ] Behavioural & social sciences [ ] Ecological, evolutionary & environmental sciences

For a reference copy of the document with all sections, see nature.com/documents/nr-reporting-summary-flat.pdf

Life sciences study design

All studies must disclose on these points even when the disclosure is negative.

Sample size	For all experiments requiring statistical analysis, we aimed to have a minimum of n = 3 biological replicates in order to derive p-values. We noted exact sizes either within the methods section or the figure legends. Sample size selection was based on similar types of experiments in

	the literature.
Data exclusions	No data was excluded from analysis.
Replication	All experiments were repeated at a minimum of 2 times will all replication attempts being successful.
Randomization	For all relevant studies/experiments, the same homogeneous sample was randomly split into either a treatment or control groups.
Blinding	Investigators were blind to primary human samples that were acquired for sequencing experiments as they were de-identified. For the various experimental/treatment studies, investigator was not blinded to group allocation because the same investigator conducted data collection and data analysis.

Reporting for specific materials, systems and methods

We require information from authors about some types of materials, experimental systems and methods used in many studies. Here, indicate whether each material, system or method listed is relevant to your study. If you are not sure if a list item applies to your research, read the appropriate section before selecting a response.

Materials & experimental systems

n/a	Involved in the study	
☐	☒	Antibodies
☐	☒	Eukaryotic cell lines
☒	☐	Palaeontology and archaeology
☐	☒	Animals and other organisms
☒	☐	Clinical data
☒	☐	Dual use research of concern
☒	☐	Plants

Methods

n/a	Involved in the study	
☐	☒	ChIP-seq
☐	☒	Flow cytometry
☒	☐	MRI-based neuroimaging

Antibodies

Antibodies used

1. α-SMA Abcam Cat# ab5694. Dilution 1:50
2. KRT14 Akoya Cat# 4450031. Dilution 1:200
3. CD4 Akoya Cat# 4550112. Dilution 1:100
4. CD31 Akoya Cat# 4150017. Dilution 1:100
5. VCAM1 Abcam Cat# ab271899. Dilution 1:100
6. LSP1 Abcam Cat# ab248682. Dilution 1:50
7. FN1 Abcam Cat# ab271831. Dilution 1:50
8. CLDN4 Abcam Cat# ab53156. Dilution 1:100
9. FOXP3 Akoya Cat# 4550071. Dilution 1:50
10. DPP4 Abcam Cat# ab225901. Dilution 1:50
11. FAP Abcam Cat# ab240989. Dilution 1:200
12. CD68 Biolegend Cat# 916104. Dilution 1:50
13. IFITM3 CST Cat# 59212. Dilution 1:50
14. CHI3L1/YKL40 CST Cat# 47066. Dilution 1:100
15. PDGFRα/β Abcam Cat# ab215978. Dilution 1:50
16. KRTDAP Atlas Cat# HPA063474. Dilution 1:50
17. CXCL12/SDF1 Abcam Cat# ab9797. Dilution 1:100
18. CD3 Akoya Cat# 4550119. Dilution 1:50
19. TROP2 Abcam Cat# ab237859. Dilution 1:50
20. KRT6 Biolegend Cat# 606102. Dilution 1:100
21. ITGAV Abcam Cat# ab271932. Dilution 1:50
22. TAGLN Abcam Cat# ab14106. Dilution 1:50
23. TNC Millipore Cat# AB19011. Dilution 1:100
24. KI-67 Akoya Cat# 4250019. Dilution 1:100
25. Collagen IV Abcam Cat# ab6586. Dilution 1:50
26. POSTN Abcam Cat# ab14041. Dilution 1:50
27. CD45 Akoya Cat# 4550121. Dilution 1:50
28. VIM Akoya Cat# 4450050. Dilution 1:100
29. MUC-1 Abcam Cat# ab28081. Dilution 1:200
30. Collagen I Abcam Cat# ab215969. Dilution 1:50
31. CD8 Akoya Cat# 4250012. Dilution 1:50
32. EpCAM Akoya Cat# 4550088. Dilution 1:50
33. MMP-9 Biolegend Cat# 819701. Dilution 0.75:200
34. HLA-DR Akoya Cat# 4550118. Dilution 1:100

35.CD11c CST Cat# 93233. Dilution 1:50

36. S100A4 Biolegend Cat# 810101. Dilution 1:100
37. GLUT1 Abcam Cat# ab252403. Dilution 1:100
38. S100A9 Proteintech Cat# 26992-1-AP. Dilution 1:100
39. CAV1 Akoya Cat# 4550084. Dilution 1:100
40. E-cadherin Abcam Cat# ab256580. Dilution 1:100
41. MPO Abcam Cat# ab221847. Dilution 1:100
42. KRT14 Biolegend Cat# 906001. Dilution 1:500
43. CD326/EpCAM – PE-Cy7 Biolegend Cat# 324222. Dilution 1:100
44. CD90/THY1 – BV711 Biolegend Cat# 328139. Dilution 1:100
45. NF-κB1 p105/p50 CST Cat# 13586. Dilution 1:50
46. anti-chicken Alexa488 secondary antibody Invitrogen Cat# A-11039. Dilution 1:500

Validation

All primary antibodies listed 1-45 have been validated by respective manufacturers.

Eukaryotic cell lines

Policy information about cell lines and Sex and Gender in Research

Cell line source(s)

The mouse basal cell carcinoma cell line ASZ001 was generated by Epstein group (So et al., Experimental Dermatology 2006). HEK293-mNoggin-Fc cells were a gift from the Hans Clevers group (Boonekamp et al., PNAS 2019). HEK293-HA-R-Spondin1-Fc (R&D Systems; 3710-001-01) and L-WRN cells (ATCC; CRL-3276) were commercially purchased.

Authentication

ASZ001 were authenticated previously by the Epstein group (So et al., Experimental Dermatology 2006). In our experiments, ASZ001 cells are monitored by their morphology as well, sequencing, and utilization of mouse specific primers. In our experiments, HEK293-mNoggin-Fc, HEK293-HA-R-Spondin1-Fc, and L-WRN cells are monitored by their morphology.

Mycoplasma contamination

No mycoplasma was detected.

Commonly misidentified lines
(See ICLAC register)

None.

Animals and other research organisms

Policy information about studies involving animals; ARRIVE guidelines recommended for reporting animal research, and Sex and Gender in Research

Laboratory animals

Mice were housed under standard conditions, and animal care was compliant with the protocols approved by the Institutional Animal Care and Use Committee (IACUC) at Stanford University. Ptch1+/-;p53f/f;K14Cre-ER mice (with or without RFPf-s-f) were generated and utilized to develop BCC tumors. Cre-mediated recombination was induced through three 4-hydroxytamoxifen injections (50µl of 5mg/ml; Sigma;H7904) completed once per day for three consecutive days. Within one day of the final dose, mice were irradiated (5.25 Gy) using an X-ray irradiator. Primary tumors developed four to six months afterward. Irradiated mice were monitored multiple times per week for signs of discomfort and distress and for tumor development. Animals persistently scratching and causing ulcerations despite analgesics and topical antibiotics were considered for euthanasia. Adult female Nod-Scid mice (7-8 weeks of age) (Jackson Laboratory, Stock number: 001303) were used as host animals for grafted tumors. We only used female mice for allografts because male mice bite the grafts. Host mice with grafted tumor cells were monitored at least 5 days a week for signs of discomfort and distress and any mice that exhibited poor grooming and health were euthanized. Tumor graft sites were closely monitored to ensure tumor burden did not exceed the maximal tumor burden at 10% of pre-inoculation body weight of each mouse. Palpable tumors were developed after 1-2 months of grafting, upon which host mice were euthanized using CO_2 and tumors were collected for analysis. All experiments were compliant with the ethics guideline.

Wild animals

None.

Reporting on sex

Adult female Nod-Scid mice (7-8 weeks of age) (Jackson Laboratory, Stock number: 001303) were used as host animals for grafted tumors. We only used female mice for allografts because male mice bite the grafts.

Field-collected samples

None.

Ethics oversight

All mouse usage (husbandry, procedures, experiments, etc.) were approved by the Institutional Animal Care and Use Committee (IACUC) at Stanford University.

Note that full information on the approval of the study protocol must also be provided in the manuscript.

Plants

Seed stocks | N/A

Novel plant genotypes | N/A

Authentication | N/A

ChIP-seq

Data deposition

☒ Confirm that both raw and final processed data have been deposited in a public database such as GEO.

☒ Confirm that you have deposited or provided access to graph files (e.g. BED files) for the called peaks.

Data access links<br>*May remain private before publication.*	https://www.ncbi.nlm.nih.gov/geo/query/acc.cgi?acc=GSE248314
Files in database submission	PBS_NFKB1 - 2 replicates (fastq, bigwig), Ligand_NFKB1- 2 replicates (fastq, bigwig).
Genome browser session<br>(e.g. UCSC)	*Provide a link to an anonymized genome browser session for "Initial submission" and "Revised version" documents only, to enable peer review. Write "no longer applicable" for "Final submission" documents.*

Methodology

Replicates	There were two experimental conditions in this CUT&RUN experiment: (1) PBS control and (2) ligand-treated mouse BCC cells. All samples were performed in 2 independent biological replicates.
Sequencing depth	Ligand_NFKB1_1 (Total read pairs: 64331244, Mapped pairs: 61210262)<br>Ligand_NFKB1_2 (Total reads pairs: 63153379, Mapped pairs: 59612000)<br>PBS_NFKB1_1 (Total reads pairs: 77788683, Mapped pairs: 66747169)<br>PBS_NFKB1_2 (Total reads pairs: 52674530, Mapped pairs: 50086892)
Antibodies	anti-NFKB1 antibody or an IgG antibody control
Peak calling parameters	Fastq files were aligned to mm10 using bowtie2 Version 2.3.4.1 (-p 4 --very-sensitive). Peak calling was carried out with MACS2 using default settings and q-value 0.05.
Data quality	Fastq files were processed with TrimGalore 0.5.0 to assess data quality.<br>MACS2 was used for peak calling using FDR threshold 0.01.<br>Ligand_NFKB1_1: 25818<br>Ligand_NFKB1_2: 24941<br>PBS_NFKB1_1: 14389<br>PBS_NFKB1_2: 6569
Software	Paired-end reads were trimmed for Illumina adaptor sequences using Trim Galore and mapped to mm10 using Bowtie2 Version 2.3.4.1 with parameters -p 4–very-sensitive. Duplicate reads were discarded with Samtools v0.1.18. MACS2 with FDR threshold 0.01 was used for peakcalling and background removal was carried out via submitting replicates to irreproducible discovery rate (IDR) filtering. Overlapping peaks from all samples were merged into a unique peak list, and raw read counts mapped to each peak [using bedtools multicov (Quinlan laboratory, University of Utah, Salt Lake City, UT)] for each individual sample were quantified. Differential analysis was performed using DESeq2.

Flow Cytometry

Plots

Confirm that:

☒ The axis labels state the marker and fluorochrome used (e.g. CD4-FITC).

☒ The axis scales are clearly visible. Include numbers along axes only for bottom left plot of group (a 'group' is an analysis of identical markers).

☒ All plots are contour plots with outliers or pseudocolor plots.

☒ A numerical value for number of cells or percentage (with statistics) is provided.

Methodology

Sample preparation	As noted in Methods section, single-cell suspensions of cells were resuspended in 2% FBS FACS buffer and then stained with appropriate antibodies at a 1:100 dilution for 60 mins at 4 °C in the dark. Antibodies used were PE/Cy7 anti-human CD326 (Biolegend; 324222) and BV711 anti-human CD90 (Biolegend; 328139). Cells were subsequently washed twice using FACS buffer and strained through a 40µm filter. Before sorting or flow, SytoxBlue (Thermo Fisher; S34857) was used for a viability dye at a 1:1000 dilution. FACS experiments were run on FACSAria II, and flow experiments were run on Symphony 2 instruments. Both instruments used the BD FACSDiva 8.0.1 software for data collection. For scRNA-Seq experiments, cells were only stained with SytoxBlue, and live cells were sorted for downstream processing and experimentation. For ApotrackerTM experiments, we used the ApotrackerTM Green reagent as per manufacturer instructions (BioLegend; 427402). FlowJo version 10.6 was used for data analysis.
Instrument	FACS experiments were run on FACSAria II, and flow experiments were run on Symphony 2 instruments. Both instruments used the BD FACSDiva 8.0.1 software for data collection.
Software	BD FACSDiva software was used for data collection on the instrument. For downstream analysis, we used FloJo v10.6.
Cell population abundance	For flow analysis experiments, cell viability was approximately 10-60% depending on the tumor.
Gating strategy	FSC-A/SSC-A gating was first used to identify cells and remove debris. Single cells were isolated by FSC-A/FSC-H gating. Live cells were then isolated via negative staining for SytoxBlue. Gating on CD90 and EPCAM were done to identifying tumor epithelial cells before ApoGreen gating. The boundaries between positive and negative staining were drawn based on a combination of using unstained controls as well as single stain controls.

☒ Tick this box to confirm that a figure exemplifying the gating strategy is provided in the Supplementary Information.
